# Tau-Reactive Endogenous Antibodies: Origin, Functionality, and Implications for the Pathophysiology of Alzheimer's Disease

**DOI:** 10.1155/2019/7406810

**Published:** 2019-08-06

**Authors:** Lenka Hromadkova, Saak Victor Ovsepian

**Affiliations:** National Institute of Mental Health, Klecany 25067, Czech Republic

## Abstract

In Alzheimer's disease (AD), tau pathology manifested by the accumulation of intraneuronal tangles and soluble toxic oligomers emerges as a promising therapeutic target. Multiple anti-tau antibodies inhibiting the formation and propagation of cytotoxic tau or promoting its clearance and degradation have been tested in clinical trials, albeit with the inconclusive outcome. Antibodies against tau protein have been documented both in the brain circulatory system and at the periphery, but their origin and role under normal conditions and in AD remain unclear. While it is tempting to assign them a protective role in regulating tau level and removal of toxic variants, the supportive evidence remains sporadic, requiring systematic analysis and critical evaluation. Herein, we review recent data showing the occurrence of tau-reactive antibodies in the brain and peripheral circulation and discuss their origin and significance in tau clearance. Based on the emerging evidence, we cautiously propose that impairments of tau clearance at the periphery by humoral immunity might aggravate the tau pathology in the central nervous system, with implication for the neurodegenerative process of AD.

## 1. Introduction

Alzheimer's disease (AD) is a progressive neurodegenerative disorder and the most common cause of dementia in the elderly population. Cognitive decline and memory loss, with related clinical symptoms affecting everyday activities, signify the advanced stage of the pathology, preceded by preclinical and mild cognitive impairment (MCI) stages. MCI is a prodromal process, characterized by the onset of the earliest cognitive symptoms including memory dysfunctions and other cognitive impairments, which in most of the cases gradually evolve into a clinical AD [1]. In-depth analysis of pathological changes and testing potential therapeutics for the treatment of MCI appears to be of major significance, offering a window for early intervention into the disease process, with currently no cure available for AD. Neurofibrillary tangles (NFTs) composed of tau protein along with senile plaques formed by amyloid beta (A*β*) peptide are the most consistent histopathological hallmark of the disease [2, 3]. Tau protein belongs to the family of microtubule-associated proteins (MAPs) and is localized predominantly in axonal projections of neurons [4]. It plays a critical role in the microtubule assembly and stabilization [5], contributing to cytoskeleton reorganization and regulation of axonal transport, via dynamic interactions with microtubules [6–9]. Under neurodegenerative conditions such as AD and other tauopathies, aggregation of pathologically modified variants of tau protein and its abnormal sorting lead to the disruption of normal neuronal functions and formation of intracellular tangles and cytotoxic soluble toxic oligomers. Therefore, tau protein is considered as one of the key ingredients involved in disease and, as such, a warranted diagnostic and therapeutic target.

Due to the critical involvement of the immune system in the pathobiology of AD, antibody-based therapies are under close scrutiny as a promising disease-modifying strategy. To this end, circulating immunoglobulins reactive with A*β* peptides and tau protein are of special research and translational interest [10]. While much progress has been made in this direction, debate persists as to whether disease-related changes in the level and activity of these antibodies are beneficial or harmful. Until recently, most of the research was focused on natural A*β* peptide-reactive antibodies [11]. Nevertheless, in clinical trials, therapeutic strategies based only on anti-A*β* treatments turned disappointing, suggesting also other players [12, 13]. Tau pathology, on the other hand, correlates better with progression of the clinical AD and neurodegenerative process and can evolve independently from A*β*-related impairments [14]. Accordingly, various anti-tau antibodies have been produced and intensively tested for their putative protective role [15, 16].

Throughout this review, we discuss emerging data on tau-reactive antibodies in circulatory systems and consider their relevance to tau homeostasis and dysregulations in AD. We highlight advances and stress the lack of consensus in the field, along with urgent need of in-depth research, to elucidate the role of humoral immunity in tau homeostasis. Finally, we discuss the possible significance of tau-reactive antibodies for countering the progression of the neurodegenerative process in AD.

## 2. Tau Protein as a Potential Immunogen

Although tau is an intracellular protein with the main localization in neuronal axons [4], it also belongs to the core CSF biomarkers of AD, with trace amounts also detected in the blood.

In the CNS, tau protein occurs in six isoforms due to alternative splicing [17] and as a natively unfolded protein which is subjected to many posttranslational modifications, mostly phosphorylation [18]. Levels of total tau (T-tau) and tau phosphorylated at threonine 181 (P-tau) are elevated in CSF of AD [T-tau: 559 (85-2782) ng/L, P-tau: 82 (17-279) ng/L] and MCI due to AD [T-tau: 582 (83-2174) ng/L, P-tau: 81 (15-183) ng/L] compared to age-matched controls [T-tau: 280 (42-915) ng/L, P-tau: 51 (16-156) ng/L] [19]. However, there are marked differences in reported absolute levels of CSF biomarkers in different studies, even with the use of the same ELISA variants for measurements [20].

Until recently, there was no possibility of measuring tau protein in peripheral fluids, due to lack of sensitive methods. Research for blood-based biomarkers of neurodegenerative diseases, however, facilitated the development of a Simoa-HD1-based novel single-molecule ELISA capable of detecting minuscule amounts of tau [21]. With this method, total tau protein levels in the plasma were quantitatively evaluated in healthy volunteers, MCI, and AD patients, revealing therein far lower concentrations (units of ng/L) as compared to the CSF [22, 23]. The amount of tau reported in patients with clinical AD was somewhat higher as compared to that in MCI and controls, although there was a considerable overlap of readouts from different groups [22, 24–26]. At this point, there is pressing need in large cohort longitudinal studies with the use of ultrasensitive assays capable of detecting not only the amounts of regular tau protein but also its fragments, as well as posttranslationally modified variants prevalent in AD. Using newly developed assay, Tatebe and colleagues [27] in a recent small-scale study demonstrate significantly higher levels of phosphorylated p181 tau protein (P-tau) in the plasma in AD patients as compared to healthy controls, a finding confirmed also by other groups [28, 29]. Intriguingly, the most toxic tau oligomers have been detected in the serum not only in AD patients but also in MCI groups and in healthy volunteers, with the lowest amounts in the MCI cohort [30]. These observations suggest that various modifications of tau protein in the periphery could serve as an antigen stimulating an immunological response, with the production of a range of tau-reactive antibodies.

Because of physiological tight protection of the brain by blood-brain barriers (BBB), an increase in the amount of neural proteins in peripheral blood circulation suggests impairments of the barriers [31]. Like AD, stroke, traumatic brain injuries (TBI), and other conditions are accompanied by the disruption of the BBB with increased permeability [32]. Under these circumstances, the loss of neurovascular defense shield unmasks self-antigens of the central nervous system, activating immune response with the production of autoantibodies in the periphery. In TBI, for example, disruption of the BBB causes a significant rise in the levels of astrocytic proteins GFAP and S100b in the plasma, which stimulates the production of anti-GFAP and anti-S100b autoantibodies of the IgG subtype [33, 34]. Disruption of the vascular barriers of the brain has been suggested to contribute to rise in the levels of tau in AD [35–38] as well as in frontotemporal dementia (FTD) [39] and Down syndrome [40]. Importantly, loss in BBB integrity occurs also in normal aging as well as in MCI [41–43], enabling the bidirectional crossing of several tau protein forms across, albeit with different kinetics [44]. Another source of peripheral tau is blood platelet cells, know also to carry trace amounts of tau protein [45]. Interestingly, an increase of high molecular weight (HMW) forms of tau and a decrease of low molecular weight tau (LMW) were reported in blood platelets of AD, compared to healthy controls [45–48]. Last but not least, in the peripheral circulation, tau protein was found in association with exosomes originated from the CNS [49–52], which have been traditionally viewed as a mechanism for tau clearance [53].

The increased levels of tau protein in the peripheral circulation of AD patients could potentially reflect two pathological processes occurring in the brain: (1) BBB disruptions and (2) extensive axonal damage with neurodegeneration. Despite the rise of the level of tau protein in the blood, as noted above, levels of tau-reactive antibodies in serum at MCI and clinical AD remain unchanged [54–57]. One possible explanation for this conundrum is that with the disruption of the BBB, newly formed tau-reactive autoantibodies may be recruited from the circulation into the brain, which could aggravate the pathological process through adding extra immunological burden, a notion supported by higher IgG reactivity within damaged neurons and NFTs in AD [58, 59]. Alternatively, in AD and other related neurodegenerative diseases, the peripheral immune system is severely compromised, contributing to the paradoxical reduction of the levels of tau-reactive antibodies in the blood of diseased subjects, as compared in controls [60]. Considering wide variations in physiological and pathological variants of tau protein, the pool of tau-reactive antibodies in circulation is likely to comprise a heterogeneous mixture against various tau epitopes with distinct characteristics, an important factor to be considered in future research of tau-reactive antibodies.

## 3. Tau-Reactive Antibodies in Circulatory Systems

The presence of tau-reactive autoantibodies of both IgG and IgM has been proven in sera and cerebrospinal fluid of AD patients as well as in healthy controls [54–57, 60–64], summarized in Table 1. Antibodies directed against tau protein are also shown in various intravenous immunoglobulin (IVIG) products and pooled immunoglobulin subtype G from large cohorts of healthy donors [64–68]. Quantitative evaluation of tau-reactive antibodies in the blood has been reported by several groups [54–57, 60–62]. Even though different tau variants have been used as antigens in ELISA reports in these publications, surprisingly, no statistically significant differences were found between healthy controls and AD patients. Only higher levels of tau-reactive antibodies were detected with respect to gender in MCI due to the AD group [56]. Klaver and colleagues demonstrated elevated titers of serum tau-reactive antibodies against tau fragment 196-207 phosphorylated at residues 199/202 in a group of MCI subjects [62]. Remarkably, in several trials, the levels of tau-reactive antibodies in the serum of AD patients were lower compared to controls and decline further with the advancement of the pathology [60].

Relatively similar levels of tau-reactive antibodies in AD and controls detected in most studies call into question their general physiological significance and potentially beneficial role under pathological circumstances. The ubiquitous occurrence of tau-reactive antibodies in sera and cerebrospinal fluid of healthy individuals and in IVIG products suggests that they are unlikely to be harmful [57, 65]. But to date, it can only be speculated whether they may have protective effects against tau pathology in AD or other tauopathies, by facilitating the clearance of misfolded tau, blocking its polymerization, or perhaps degrading tau aggregates [69]. It is worth stressing that specific tau-reactive antibodies may be also generated in response to the presentation of tau as new self-antigen, which might contribute to the immune imbalance in AD, and potentially lead to neuroinflammatory response, aggravating the progression of the disease.

Overall, while overwhelming data indicate the presence of tau antibodies in circulatory liquids in the brain and at the periphery, debate persists over their origin and relevance to normal physiology and pathobiology of AD. Whether these immunoglobulins are naturally occurring antibodies, mostly with the poly/cross-reactive profile, or whether they are generated as more specific antibodies after the exposure of tau protein as an antigen with newly exposed epitopes to the peripheral immune system remains to be determined. As discussed below, an in-depth analysis of these important issues has led recently to several interesting discoveries, bringing more clarity in complex affairs of the immune system and the brain under healthy and diseased conditions.

## 4. Naturally Occurring Tau-Reactive Antibodies

The generalized assumption that low-affinity polyreactive natural anti-tau antibodies have beneficial functions while high-affinity nonreactive autoantibodies are harmful, while instructive, requires rigorous experimental validation. With growing recognition of major immunogenic components in AD and other chronic neurodegenerative diseases and interest in immunotherapy, there is a pressing need in the research of naturally occurring endogenous antibodies directed to brain antigens [59, 70]. Whether peripheral antibodies reactive with tau protein belong to natural antibodies with more beneficial function or they are more likely to be formed due to autoimmunity process is still unknown.

The prevalence of tau-reactive natural human antibodies shown recently in peripheral circulation presents a major advance [57]. Their occurrence in circulation from early childhood suggests the independence of this process from the exogenous antigen and, as such, perhaps acting as a part of the innate immunological repertoire, possibly involved in the regulation of tau homeostasis under physiological and pathological conditions. Thus, tau-reactive antibodies could be a part of a pool of naturally occurring antibodies, germline gene-coded antibodies produced independently of foreign antigens, which constitute approximately two-thirds of total serum immunoglobulins. As they are not subjected to affinity maturation and thus contain none or very few somatic mutations, they are associated with polyspecific (polyreactive) and variable, predominantly low, antigen-binding affinities [71, 72] and are assumed to have many crucial physiological roles and contribute significantly towards the maintenance of immune homeostasis [73–76]. In 2018, the first report of the longitudinal evolution of tau antibody levels in the serum of AD and controls was published [60]. Surprisingly, a gradual decrease in serum anti-tau antibodies in the course of AD compared to controls was found, which may reflect a progressive loss of tau-reactive antibodies with possible immunomodulatory functions during the AD-linked neurodegeneration. This observation, however, requires independent verification, using a larger collection of samples.

Beside predominantly beneficial natural antibodies, autoantibodies causing a range of chronic autoimmune disorders can be also produced, typically due to dysregulations of the immune system [77, 78]. These antibodies are almost exclusively monoreactive with higher affinities to particular self-antigens. Several hypotheses, explaining how a part of antibody portfolio switches into pathogenic autoantibodies, have been suggested [79, 80], with the most acknowledged one known as the autoimmune hypothesis of AD [58, 81]. In brain regions most severely affected by AD, Ig-positive neurons are in excess, as compared to age-matched controls [59]. Moreover, many of these Ig-positive neurons show morphological signs of neurodegeneration and express markers of classical complement pathway and apoptosis [82]. Blennow and colleagues demonstrated the increased intrathecal synthesis of either IgM or IgG in some AD patients, which suggested the activation of immune response by the degenerative process [83]. Recently, increased intrathecal synthesis of anti-tau antibodies was shown in multiple sclerosis [55] and confirmed in AD [61]. Elevated intrathecal synthesis of anti-tau antibodies, reflecting the activation of the specific humoral immune response to extracellular tau released by the neurodegenerative process, can be perceived as an autoimmune response within the CNS. Whether the pool of anti-tau antibodies produced in the brain exacerbates the pathogenic process or counters it remains to be established. With the progression of AD, the peripheral tau protein levels also become higher, suggesting progressive impairment of the BBB, with tau released in peripheral circulation, where it might serve as an antigen in the formation of tau-reactive antibodies.

To summarize, antibodies reactive with tau protein are more likely to be represented by a mixture of both, the natural antibodies without antigen-dependent maturation and more specific antibodies formed after the exposure to tau as an immunogen. The crucial question that remains to be addressed is whether the antibodies against pathological tau forms may help their clearance or whether they bind with them and thus contribute towards the inflammation and harmful immunological response unfolding in AD.

## 5. Reactivity of Circulating Anti-Tau Antibodies

**Figure 1:**
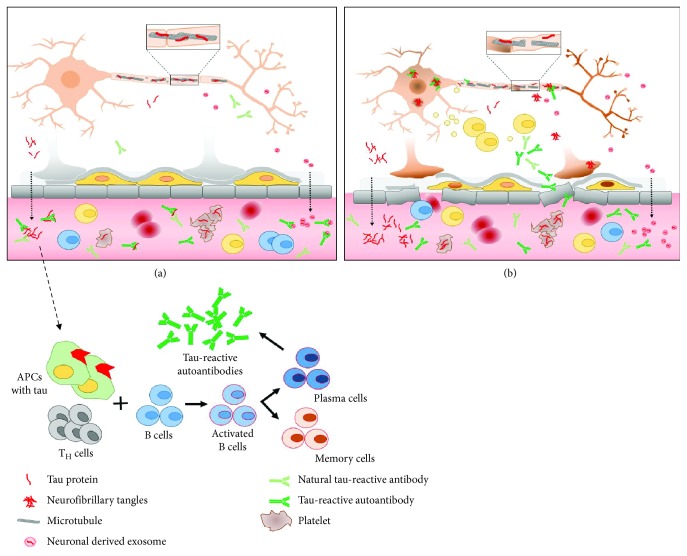
Schematic illustration of the concept of circulating tau-reactive antibodies. Tau protein is present in blood circulation where it may serve as an immunogen for the production of tau-reactive autoantibodies. In the periphery, there is a repertoire of natural antibodies, which may cross-react with tau and which help to maintain immune homeostasis. (a) illustrates physiological condition where the blood-brain barrier (BBB) is intact and the small amounts of tau efflux to the periphery is balanced by tau clearance and degradation. (b) illustrates pathological condition, such as in AD. Under this circumstance, BBB is impaired and, as a result of many large immunogenic molecules, including tau protein, leaks across barriers into peripheral circulation and vice versa, into the CNS. The latter is thought to aggravate the neuroinflammation and neurodegenerative process in AD. APC = antigen-presenting cell.

Using circulating tau-reactive antibodies from the peripheral system, it was shown that these antibodies can recognize abnormally modified tau forms and vary in a great deal in their characteristics [64, 67]. These findings are in general agreement with miscellaneous and highly complex transformation, which tau protein undergoes throughout the initial aggregation process and formation of paired helical filaments (PHFs) followed by more dynamic changes involving truncation and phosphorylation during the polymerization process (the ordered series of events in NFTs evolution are described at [84–88]). The same processes also lead to the formation of oligomeric tau variants, which are thought to be the most toxic species, contributing towards spreading of tau pathology and neurodegeneration [89]. In light of this, an in-depth study with the characterization of the activity of circulating tau-reactive antibodies against physiological and pathological tau forms is well warranted. Our preliminary data showed that tau-reactive antibodies extracted from AD plasma interact with monomeric tau forms, both recombinant and derived from native brain homogenates. In contrary, antibodies isolated from IVIG and pooled from the plasma of healthy controls showed stronger reactivity with recombinant fragmented tau (155-421 aa) protein and with more aggregated forms presented in brain homogenates of AD [64, 67]. So far, these observations support the notion that the humoral immune system may be involved in controlling the occurrence of abnormal tau protein forms under physiological conditions. Overall, it emerges that tau protein leaking from the brain into the peripheral circulatory system is subjected to fast degradation and elimination by endogenous tau-reactive antibodies, a process which seems to be compromised in AD (Figure 1). The latter is supported by recent data showing that enhancement of tau clearance in the periphery is an effective means for ameliorating tau pathology in the central nervous system of murine AD models and in human brain [90].

## 6. IVIG for AD Immunotherapy

Aberrations of tau protein conformation play a major role in the pathobiology of AD; therefore, antibody-based immunotherapy directed against structurally modified and aggregated tau variants presents an attractive therapeutic strategy [10]. The pathological changes in tau protein occurring during MCI and AD progression are, however, highly complex and not strictly tied to specific molecular conformation or mechanism. The therapeutic use of IVIG products prepared from the plasma of healthy controls enriched with a wide range of natural antibodies is therefore highly warranted, offering a miscellaneous solution. Over recent years, numerous beneficial immunomodulatory and anti-inflammatory effects of IVIG have been documented upon their use for the treatment of some immune-mediated neurological disorders [91–93]. IVIGs have been also tested in several clinical trials with AD and MCI patients. Unfortunately, the phase III clinical trials have not fulfilled positive expectations staged by promising results of the initial phase I and II trials [94–96]. Despite the disappointing outcome so far, several IVIG clinical trials are nevertheless currently ongoing [97, 98]. Intense research is also on its way towards a better characterization of antibodies presented in IVIGs that are specifically directed against proteins relevant to the pathobiology of AD. Based on these studies, it is recommended to use disease-specific IVIG preparations enriched with target-specific antibodies [70, 99].

To the best of our knowledge, to date, only five reports analyzed IVIG products containing tau-reactive antibodies [64–68], summarized in Table 1. After the pioneering report presenting the first evidence for the presence of natural antibodies directed to recombinant human full-length tau in three IVIG products [65], studies have been undertaken to show also the enrichment of IVIGs with antibodies against various tau regions [66] and AD-specific phosphorylated tau forms [67]. In all these reports, tested IVIG products contained autoantibodies reactive to tau antigens, with their levels significantly varying among different IVIG products. The latter possibly reflect variations in specific antibody concentrations amongst donor pools used for plasma collection, as well as the procedures applied for antibody isolation and purification [65, 100]. The lower *in vitro* binding affinity for antibodies might also contribute to the general variability of the research outcome [101]. Finally, polyvalent binding of immunoglobulins needs to be taken into account, to accurately determine the concentrations of a specific natural antibody in complex samples such as a serum, plasma, and IVIG [102].

## 7. Closing Remarks

Throughout this study, we reviewed recent evidence concerning the origin and functionality of tau-reactive antibodies in the circulatory systems of the brain and periphery and discussed their relevance to pathophysiology and therapy of AD. Although much progress has been made in this direction, numerous basic and translational questions remain unanswered, requiring new thinking and in-depth research. There is also a major need in new assays with higher sensitivity, for the detection and quantification of a wide variety of tau proteins, as well as antibodies reactive to tau, which will allow better substantiation of the research and therapeutic interventions, including the use of IVIG immunotherapy. Important lessons should be learned from disappointing outcomes in clinical trials, which testify the incompleteness of our understanding of the biology of autoantibodies and pathophysiology of AD. Arguably, the most exciting outcome of recent studies is the recognition that throughout evolution, a powerful natural mechanism has been set to maintain the homeostasis of tau protein and facilitate its clearance in peripheral circulation when in excess, which can be exploited and guided for therapeutic benefit. With incentives getting higher for effective AD-modifying therapies, reviewed herein are advances in tau-reactive antibodies and their role in tau clearance is likely to become an important part of gradually emerging complex solution to this debilitating disease in the foreseeable future.

## Figures and Tables

**(a):** 

Serum and cerebrospinal fluid					
Detection method	Tau antigen	Body fluid	Group of participants	Main results	References
EIA	Bovine tau	CSF, serum	Controls, AD, PD, MS, ALS, ON, OND, SMN/CIDP, GBS, VD	Anti-tau Abs detected above assay cut-off value only in sera of two controls (2/44) and one ALS (1/26) Calculations of intrathecal synthesis	Terryberry et al. [63]
ELISA (IgG, IgM)	Full-length human tau (1-441 aa) Phosphorylated tau peptide (195-213 aa, pS202/pT205)	CSF, serum	Controls, AD, OND	Higher IgM titers of serum Abs against phosphorylated tau peptide detected in the AD group compared to the controls	Rosenmann et al. [54]
ELISA	Bovine tau	CSF, serum	Controls, MS	CSF anti-tau Abs levels: lower in MS patients receiving therapy than those without treatment Measurements of avidity, calculations of intrathecal synthesis	Fialova et al. [55]
ELISA	Bovine tau	CSF, serum	Controls, MS	Measurements of avidity	Fialova et al. [104]
ELISA	Bovine tau	CSF, serum	Controls, AD, OD, IC	Calculations of intrathecal synthesis AD group: higher intrathecal anti-tau Abs than the other groups	Bartos et al. [61]
ELISA	Full-length human tau (1-441 aa)	Serum	Controls, PD, bvFTD	PDD group: decreased serum anti-tau Abs compared to PDND	Kronimus et al. [105]
ELISA (IgG, IgM)	Tau peptide (196-207 aa): nonphosphorylated and phosphorylated at pS199/pS202	Serum	AD, MCI, NCI	Anti-tau IgG Abs increased in MCI vs. AD and NCI	Klaver et al. [62]
ELISA, WB	Full-length human tau (1-441 aa) Truncated form Tau155-421 Brain homogenates	Pooled plasma	Controls, AD	Different reactive profiles of anti-tau Abs isolated from pooled plasma samples (isolation procedure described in [106])	Krestova et al. [64]
ELISA	Full-length human tau (1-441 aa) Truncated form Tau155-421 Bovine tau	CSF, serum	Controls, MCI, MCI-AD, AD	Serum anti-tau Abs against bovine tau: lower in female MCI-AD vs. controls, higher in male MCI-AD vs. controls Calculations of intrathecal synthesis	Krestova et al. [56]
ELISA	Full-length human tau (1-441 aa)	Serum	Children, adults	No significant differences in anti-tau Abs levels	Kuhn et al. [57]
ELISA	Bovine tau	Serum	Controls, AD	Lower anti-tau Abs in AD vs. controls, and their decrease over time	Bartos et al. [60]

**(b):** 

Intravenous immunoglobulin G (IVIG) preparations				
Detection method	Tau antigen	Supplier of IVIG	Main results	Reference
ELISA	Full-length human tau (1-441 aa)	Gamunex (Talecris Biotherapeutics Inc., Research Triangle Park, NC) Gammagard (Baxter Healthcare Corp., Westlake Village, CA) Flebogamma (Grifols Biologicals Inc., Los Angeles, CA)	Anti-tau bs detected in all three IVIG products Mean Ab levels: 3.13 *μ*g/mL for Gammagard, 2.48 *μ*g/mL for Gamunex, 1.23 *μ*g/mL for Flebogamma	Smith et al. [65]
ELISA	Full-length human tau (1-441 aa) Tau peptides: 45–73 aa, 244–274 aa, 275–305 aa, 306–336 aa, 337–368 aa, 422–441 aa	Gamunex (Talecris Biotherapeutics Inc., Research Triangle Park, NC) Gammagard (Baxter Healthcare Corp., Westlake Village, CA) Flebogamma (Grifols Biologicals Inc., Los Angeles, CA)	Anti-tau Abs detected in all three IVIG products Differences in reactivity against various tau peptide sequences among IVIG products	Smith et al. [66]
ELISA	Tau peptide of 196-207 aa, nonphosphorylated and phosphorylated at pS199/pS202	Gamunex-C (Grifols Therapeutics Inc., Research Triangle Park, NC) Gammagard Liquid (Baxter Healthcare Corp., Westlake Village, CA) Gammaked (Talecris Biotherapeutics Inc., Research Triangle Park, NC) Flebogamma (Grifols Biologicals Inc., Los Angeles, CA) Privigen (CSL Behring AG, Bern, Switzerland) Octagam (Octapharma AB, Stockholm, Sweden)	Anti-tau Abs present in all IVIG products Differences in reactivity among IVIG products	Loeffler et al. [68]
ELISA, WB	Full-length human tau (1-441 aa) and truncated forms Tau155-421, Tau13-391, nonphosphorylated and phosphorylated	Flebogamma (Grifols Biologicals Inc., Los Angeles, CA)	Basic characterization of anti-tau Abs isolated from IVIG product	Hromadkova et al. [67]
ELISA, WB	Full-length human tau (1-441 aa) and truncated form Tau155-421, nonphosphorylated and phosphorylated Brain homogenates	Flebogamma (Grifols Biologicals Inc., Los Angeles, CA)	Basic characterization of anti-tau Abs isolated from IVIG product	Krestova et al. [64]

Abbreviations: aa: amino acids; Ab: antibody; AD: Alzheimer's disease; ALS: amyotrophic lateral sclerosis; bvFTD: behavioral variant of frontotemporal dementia; CIDP: chronic inflammatory demyelinating polyradiculoneuropathy; CSF: cerebrospinal fluid; EIA: enzyme immunoassay; ELISA: enzyme-linked immunosorbent assay; GBS: Guillain-Barre' syndrome; IC: neuroinflammatory diseases; MCI: mild cognitive impairment (MCI-AD: MCI due to AD); MS: multiple sclerosis; NCI: no cognitive impairment; OD: other dementias; ON: optic neuritis; OND: other neurologic disease; PD: Parkinson disease (PDD: PD with dementia; PDND: nondemented PD); SMN: sensorimotorneuropathy; VD: vascular dementia; WB: western blot.
